# Assessing the Impacts of Experimentally Elevated Temperature on the Biological Composition and Molecular Chaperone Gene Expression of a Reef Coral

**DOI:** 10.1371/journal.pone.0026529

**Published:** 2011-10-27

**Authors:** Anderson B. Mayfield, Li-Hsueh Wang, Pei-Ciao Tang, Tung-Yung Fan, Yi-Yuong Hsiao, Ching-Lin Tsai, Chii-Shiarng Chen

**Affiliations:** 1 National Museum of Marine Biology and Aquarium, Checheng, Pingtung, Taiwan, R.O.C.; 2 Graduate Institute of Marine Biotechnology, National Dong-Hwa University, Checheng, Pingtung, Taiwan, R.O.C.; 3 Department of Biology, University of Louisiana, Lafayette, Lafayette, Louisiana, United States of America; 4 Graduate Institute of Biodiversity and Evolution, National Dong-Hwa University, Checheng, Pingtung, Taiwan, R.O.C.; 5 Department of Marine Resources, National Sun Yat-Sen University, Kaohsiung, Taiwan, R.O.C.; Lehigh University, United States of America

## Abstract

Due to the potential for increasing ocean temperatures to detrimentally impact reef-building corals, there is an urgent need to better understand not only the coral thermal stress response, but also natural variation in their sub-cellular composition. To address this issue, while simultaneously developing a molecular platform for studying one of the most common Taiwanese reef corals, *Seriatopora hystrix*, 1,092 cDNA clones were sequenced and characterized. Subsequently, RNA, DNA and protein were extracted sequentially from colonies exposed to elevated (30°C) temperature for 48 hours. From the RNA phase, a heat shock protein-70 (*hsp70*)-like gene, deemed *hsp/c*, was identified in the coral host, and expression of this gene was measured with real-time quantitative PCR (qPCR) in both the host anthozoan and endosymbiotic dinoflagellates (genus *Symbiodinium*). While mRNA levels were not affected by temperature in either member, *hsp/c* expression was temporally variable in both and co-varied within biopsies. From the DNA phase, host and *Symbiodinium hsp/c* genome copy proportions (GCPs) were calculated to track changes in the biological composition of the holobiont during the experiment. While there was no temperature effect on either host or *Symbiodinium* GCP, both demonstrated significant temporal variation. Finally, total soluble protein was responsive to neither temperature nor exposure time, though the protein/DNA ratio varied significantly over time. Collectively, it appears that time, and not temperature, is a more important driver of the variation in these parameters, highlighting the need to consider natural variation in both gene expression and the molecular make-up of coral holobionts when conducting manipulative studies. This represents the first study to survey multiple macromolecules from both compartments of an endosymbiotic organism with methodologies that reflect their dual-compartmental nature, ideally generating a framework for assessing molecular-level changes within corals and other endosymbioses exposed to changes in their environment.

## Introduction

Coral reefs are currently threatened by an onslaught of anthropogenic stressors, with global climate change (GCC) being likely the most significant in both geographic scale and potential for devastation [1]. Rising temperatures, in particular, are likely to negatively impact reef-building corals [2], as these anthozoan-dinoflagellate (genus *Symbiodinium*) endosymbioses readily disintegrate (“bleach”) under prolonged exposure to thermal stress [3] and thus appear to live near the upper threshold of their thermotolerance [4]. Due to the likelihood of increasingly frequent and severe bleaching events occurring in the near future [5], there is an urgent need to better understand the stress response of reefs corals, particularly at the sub-cellular level [6]–[7].

The common, Pacific pocilloporid, *Seriatopora hystrix*, would make an excellent candidate for studying the effects of GCC and other anthropogenic insults, given its widespread distribution across the Indo-Pacific [8], high growth rate [9], and limited ability to tolerate increases in temperature over the average summer monthly mean [10]. Specifically, there is an interest in understanding the early-stage (minutes to hours), sub-cellular response of *S. hystrix* to thermal stress such that molecular biomarkers could be developed which would allow for health assessment on a proactive timescale. Currently however, the transcriptomic resources for *S. hystrix* are poor, a limitation we sought to overcome by sequencing 1,092 cDNA clones from which we could identify and characterize potential biomarkers for assessing the molecular-level, pre-bleaching effects of elevated temperature on this coral.

Most eukaryotes respond to thermal stress by translating molecular chaperones such as heat shock protein-70 (HSP70), which refold denatured proteins and/or prevent their aggregation [11]. Thus, the mRNA encoding this protein, *hsp70*, is typically produced at elevated quantities after exposure to thermal stress [12]. Unfortunately, studies of both this molecule and other molecular chaperones in coral [13]–[25] have either failed to discriminate between host and *Symbiodinium* homologs or neglected to account for the biological composition or quality of the samples, thus not allowing for accurate comparisons of gene or protein expression. This inherent bias in working with dual-compartmental organisms applies not only to candidate gene and protein studies, but also to those targeting thousands of molecules, such as microarray and next generation sequencing-based endeavors. Given the importance of HSP70 in cellular adaptation to thermal stress, it would be interesting to understand its compartment-specific mRNA expression patterns in corals exposed to elevated temperatures over a pre-bleaching timescale. Therefore, after identifying a *hsp70*-like homolog, *hsp/c*, from the host coral, *S. hystrix* colonies were exposed to either control (27°C) or elevated (30°C) temperature over 48 hours, and both coral and *Symbiodinium hsp/c* mRNA expression were measured with real-time quantitative PCR (qPCR) with the expectation that mRNA expression of this ortholog would co-vary across compartments and demonstrate induction in samples of the 30°C treatment.

Given the endosymbiotic nature of reef corals, it is possible that not only physiology [26]–[27], but also biological composition [28], could change either over time or in response to elevated temperature. As such, DNA and protein were also extracted from each of 90 samples in order to make conjectures as to the molecular composition of the assayed samples. A variety of parameters, including RNA/DNA and protein/DNA ratios, total holobiont soluble protein (THSP), and host and *Symbiodinium* genome copy proportions (GCPs) were calculated, and collectively, it was hypothesized that this three-tiered (RNA, DNA, and protein) approach would generate a more comprehensive snapshot of the sub-cellular composition and response of this ecologically-important coral to an environmentally relevant increase in seawater temperature.

## Results

### cDNA library

**Figure 1:**
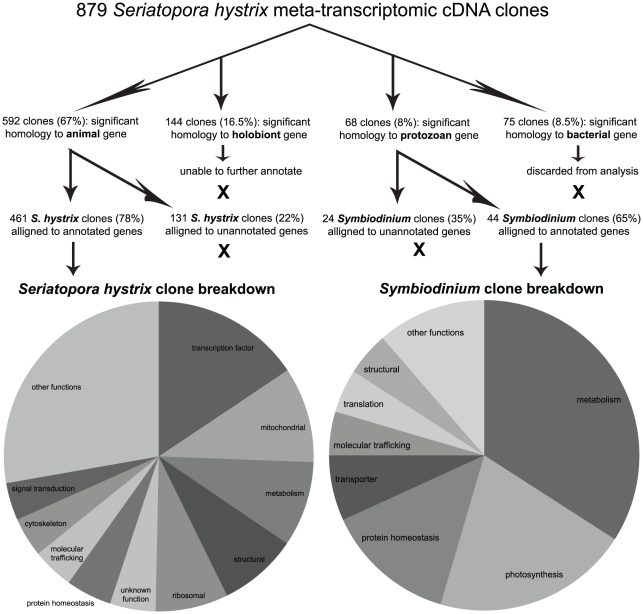
Schematic of cDNA library analysis and pie chart of results. A detailed list of select genes can be found in Table 1 (*Symbiodinium*) and Table S1 (coral).

Of the 1,092 sequenced cDNAs, 929 (85%) passed the quality control screening, and of these 929 clones, 879 (95%) could be assigned to one of the following categories with tBLASTx at an e<10^−6^ stringency (Fig. 1); bacteria (75 clones, 8.5%), animal (592 clones, 67%), protozoan (68 clones, 8%), or “holobiont” (of either animal or protozoan origin, 144 clones, 16.5%). Clones assigned to these categories were assumed to be from bacteria, coral, *Symbiodinium*, or either *Symbiodinium* or coral, respectively. As whole coral tissues were used for RNA extractions, it is conceivable that the bacterial sequences were from bacteria residing within or on the coral. However, as culture contamination cannot be conclusively ruled out, the bacterial clones were excluded from analysis, and only the remaining 804 clones, of which 73.5%, 8.5%, and 18% were from the coral (NCBI accession JN244384–JN244441, JN244443–JN244531, JN244533–JN244550, JN244552, JN244554–JN244616, JN244618–JN244619 [annotated clones] and JN600121–JN600257 [unannotated clones]), *Symbiodinium* (JN244619–JN244649 [annotated clones] and JN599978–JN600000 [unannotated clones]), and the holobiont (JN600001–JN600120), respectively, were considered for further analysis. The holobiont clones were deduced based on significant alignments to cDNA sequences from *P. damicornis* [29], yet could not be further resolved as to being of anthozoan or dinoflagellate origin due to the mixed-organismal nature of the *P. damicornis* tissues used in the library preparations of [29].

Of the 592 presumed coral clones (of which 184 within 19 functional categories are presented in Table S1), 461 (78%) were highly similar (e<10^−6^) to published and annotated sequences in the NCBI “nr” or “est-others” databases and were thus ascribed putative identities. Of these 461 clones, 439 (95%) were identified as encoding proteins whose function had been at least partially characterized. Clones reflecting genes encoding transcription factors (15.6%), mitochondrial proteins (10%), and proteins involved in metabolism (8.9%), structure (8.2%), and ribosomal assembly (7.6%) were the most abundant (pie chart of Fig. 1), though a diverse array of genes was identified across 25 functional categories.

Of the 68 deduced *Symbiodinium* clones, 44 (65%) were highly similar (e<10^−6^) to published and annotated sequences in either the NCBI “nr” or “est-others” databases and were therefore ascribed putative identities. Thirty of these are presented in Table 1. These 44 clones were sorted into 12 functional categories, 8 of which are found in the pie chart of Fig. 1. Metabolism (34%), photosynthesis (20.5%) and protein homeostasis (13.6%) were the dominant functional groups.

**Table 1:** *Symbiodinium* cDNA clones representing a diversity of functional categories.

*Symbiodinium* sp. gene	NCBI accession	Top tBLASTx hit organism	e value	clones (#)
**CYTOSKELETON**				
actin	JN244620	*Symbiodinium* sp.	0	1
**DNA SYNTHESIS**				
ribonucleotide-diphosphate reductase	JN244621	*Neospora caninum*	10^−130^	1
**METABOLISM**				
glyceraldehyde 3-phosphate dehydrogenase	JN244622	*Symbiodinium muscatinei*	0	2
NAD-dependent alcohol dehydrogenase	JN244623	*Euglena gracilis*	10^−157^	2
octopine/nopaline dehydrogenase	JN244624	*Chlamydomonas reinhardtii*	10^−102^	2
phosphoglycerate mutase	JN244625	*Pfiesteria piscicida*	10^−89^	2
fructose-bisphosphate aldolase	JN244626	*Heterocapsa triquetra*	10^−175^	1
aldehyde dehydrogenase	JN244631	*Syntrichia ruralis*	10^−174^	1
cytochrome b	JN244632	*Symbiodinium goreaui*	10^−89^	1
cyclophilin-like	JN244629	*Pfiesteria piscicida*	10^−76^	1
cytochrome c oxidase subunit III	JN244627	*Karlodinium micrum*	10^−52^	1
acyltransferase-like	JN244630	*Pfiesteria piscicida*	10^−50^	1
pectinacetyltransferase-family	JN244628	*Tetrahymena thermophila*	10^−35^	1
**PHOTOSYNTHESIS**				
peridinin-chlorophyll a-binding protein	JN244633	*Symbiodinium* sp.	10^−119^	3
light-harvesting protein	JN244634	*Symbiodinium* sp.	10^−180^	1
photosystem I subunit III*	HM156699	*Symbiodinium* sp.	10^−135^	1
photosystem II	JN244635	*Heterocapsa triquetra*	10^−43^	1
**PROTEIN HOMEOSTASIS**				
cysteine protease	JN244637	*Emiliania huxleyi*	10^−23^	3
RING finger protein	JN244640	*Perkinsus marinus*	10^−38^	1
ubiquitin-conjugating enzyme family	JN244639	*Naegleria gruberi*	10^−27^	1
**STRESS**				
ascorbate peroxidase*	HM156698	*Symbiodinium* sp.	10^−176^	1
**STRUCTURAL**				
ankyrin repeat protein	JN244641	*Trichomonas vaginalis*	10^−23^	2
**TRAFFICKING**				
ADP-riboslyation factor 1	JN244642	*Pfiesteria piscicida*	10^−62^	1
kinesin motor protein	JN244643	*Tetrahymena thermophila*	10^−24^	1
**TRANSCRIPTION**				
TU502 regulator of chromosome condensation	JN244644	*Cryptosporidium hominis*	10^−154^	1
**TRANSLATION**				
RNA-binding protein	JN244646	*Perkinsus marinus*	10^−17^	1
elongation factor 1α	JN244647	*Heterocapsa triquetra*	0	1
**TRANSPORTER**				
nitrate transporter*	HM147134	*Symbiodinium* sp.	0	1
ATP binding cassette (ABC) transporter	JN244648	*Perkinsus marinus*	10^−39^	1
drug/metabolite transporter	JN244649	*Micromonas* sp.	10^−11^	1

Genes from which real-time quantitative PCR assays (Table S2) were developed are indicated by an “*.”

### Molecular chaperone isolation

**Figure 2:**
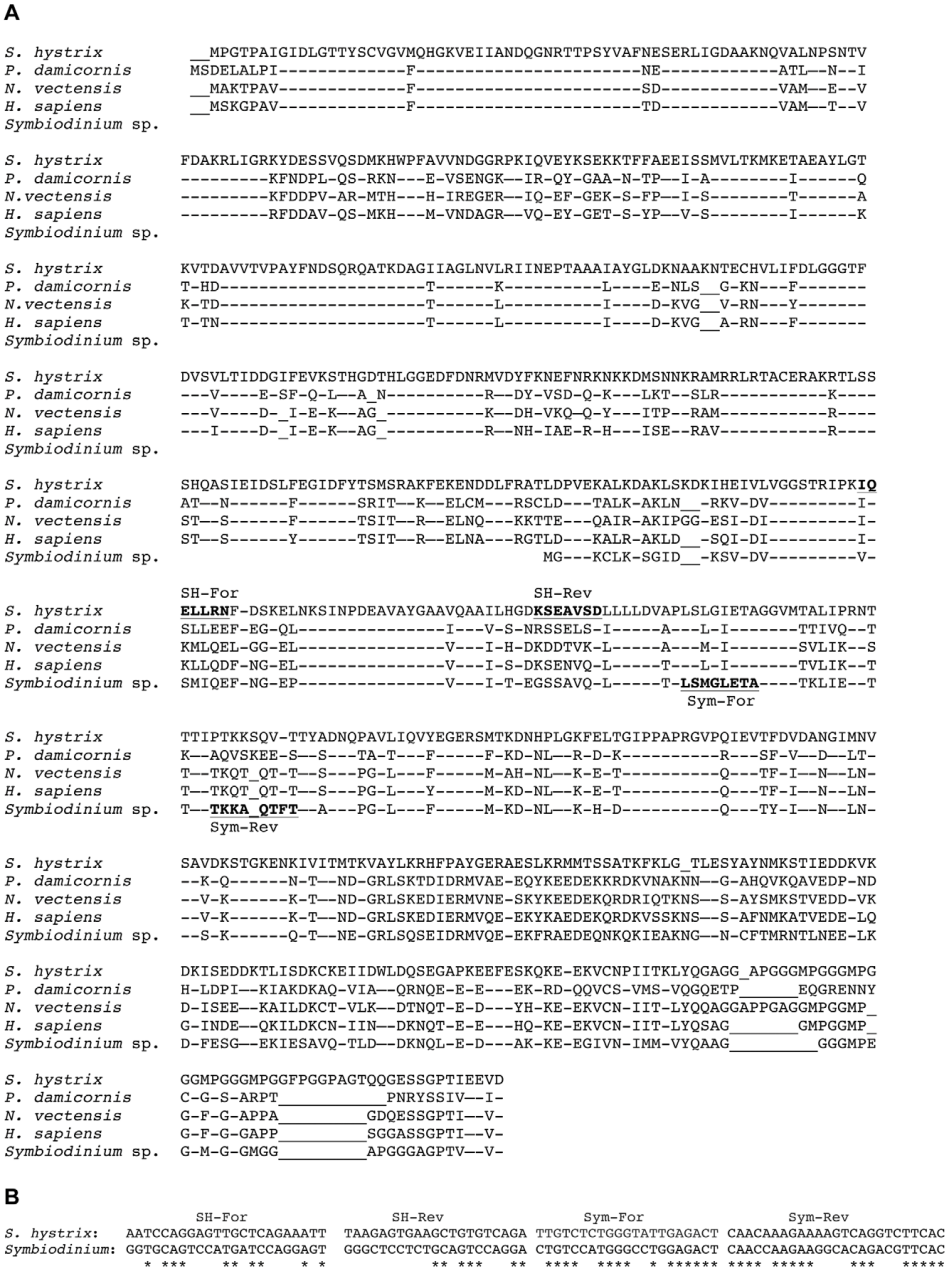
HSP/C protein alignment and primer design. (A) Protein alignment of conceptually translated host *Seriatopora hystrix* HSP/C protein with selected orthologs from the coral *Pocillopora damicornis* (BAD89541), sea anemone *Nematostella vectensis* (XP_001629343), *Homo sapiens* (NP_006588) and *Symbiodinium* sp. (clade C3, ABA28988 [partial cDNA]). Conserved residues are indicated by dashes (−), and inserts are denoted by underscores (“_”). Regions from which qPCR primers for both host and *Symbiodinium* were designed are underlined in bold font. Individual homology searches (tBLASTx) for each of these sequences returned a mix of both constitutive and inducible isoforms from a variety of taxa, disabling the ability to conclusively deduce the coral protein function solely from homology. The notable dissimilarity between the deduced *S. hystrix* protein and those of *Symbiodinium*, *P. damicornis*, *N. vectensis* and *H. sapiens* across the ∼50 C-terminal amino acids could suggest a sequencing error in the corresponding 3′ end of the gene, and, although the sequence was confirmed by two reads in each the 5′ and 3′ directions, qPCR primers were deliberately designed outside of this potentially problematic region. (B) While the region of the *hsp/c* gene targeted for expression analysis shows strong homology at the peptide level between the host coral anthozoan and its *Symbiodinium* dinoflagellates, the corresponding nucleic acid sequences from which the primers were designed demonstrate markedly less conservation (“*” denote conserved bases); the host-based *hsp/c* forward (“SH-For”) and reverse (“SH-Rev”) primers are 44 and 33% similar, respectively, to the corresponding region of the *Symbiodinium* gene. However, *Symbiodinium*-based forward (“Sym-For”) and reverse (“Sym-Rev”) primers are 71 and 74% similar, respectively, to the coral sequence, and thus a high (62.5°C) annealing temperature in the qPCR was utilized to ensure specificity.

Given that partial *Symbiodinium hsp70*-like homologs have been identified in previous studies [30]–[31], a local tBLASTx of a putative *Symbiodinium hsp70* cDNA (NCBI accession: ABA28988) was conducted against the 461 coral clones, and a potential ortholog was identified. The 2,258 base pair (bp) coral clone was comprised of a 56 bp 5′ untranslated region (UTR) followed by a 1,991 bp open reading frame (ORF) that encoded a 663 amino acid (aa) protein (Fig. 2A). This protein demonstrated significant homology to uncharacterized heat shock protein/cognate-like homologs from another pocilloporid coral, *Pocillopora damicornis*, the sea anemone *Nematostella vectensis*, and *Homo sapiens* (Fig. 2A). The ORF was followed by an approximately 175 bp 3′ UTR and then a poly-A tail, signifying that the gene had been sequenced to completion. Across the 320 aa of the partial *Symbiodinium* HSP70 protein, 62% of the residues were conserved between the hypothetical coral and dinoflagellate proteins.

The function of neither the conceptually translated protein encoded by the full-length host coral gene isolated herein nor the hypothetical protein translated from the partial *Symbiodinium* mRNA could be deduced from the sequences alone, given that top-ranking BLAST hits of both orthologs included both heat-inducible (heat shock proteins [HSPs]) and constitutively expressed (heat shock cognates [HSCs]) molecular chaperone isoforms (data not shown). As these isoforms are typically ∼85% similar at the aa level [32], qPCR primers were designed for both coral (Fig. 2B) and *Symbiodinium* [31] orthologs, deemed *hsp/c*, in order to attempt to utilize the mRNA expression patterns across an acute temperature stress exposure as a means of confirming which of the two isoforms each gene ortholog from each compartment was likely to encode. Briefly, if the heat-inducible isoform-encoding mRNAs (i.e., *hsp70*) were isolated, a significant induction to thermal stress was expected, whereas mRNAs encoding constitutively expressed isoforms (i.e., *hsc70*) would remain at statistically similar levels across both treatments.

### Field and experimental data

**Figure 3:**
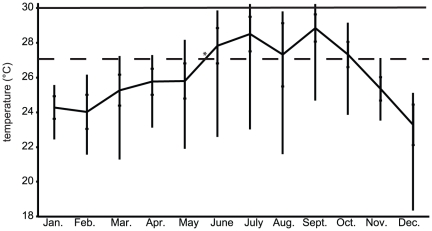
Temperature variation in 2009 at Houbihu, Southern Taiwan. Mean values are connected by the solid line, and error bars span the range for each month. The standard deviations are represented by internal hatches. The “*” denotes the approximate time at which the colonies were transplanted from Houbihu to Taiwan's National Museum of Marine Biology and Aquarium, and the dotted line (“- - -”) reflects both the acclimation and control temperature (∼27°C) prior to and during experimentation, respectively. Similarly, the solid line (30°C) reflects both the previously determined, stress-inducing temperature [27], as well as the elevated temperature used during the 48 hour experiment conducted herein.

The seawater temperature at Houbihu in 2009 (Fig. 3) fluctuated from a low of 18°C (occurring on two days in December) to a high of 30.2°C, which occurred on two days in July (logged over two consecutive hours on both days) and three days in September (logged over three consecutive hours on each day). The average annual temperature was 26.1°C, and the largest intra-month temperature variation was 8.2°C, which occurred in August, the month during which upwelling events occurred most frequently. Given both the uncommon nature of prolonged exposure to 30°C in nature and previous data on the thermal sensitivity of *S. hystrix* [27], 30°C was chosen to serve as the experimentally elevated temperature at which to expose *S. hystrix* colonies in an attempt to induce a sub-cellular thermal stress response.

During the 48 hour thermal stress experiment, aquarium temperatures converged around their target values, with averages of 27.3°C (+/−0.3°C [standard deviation of the mean]) and 30°C (+/−0.6°C) for the control and elevated temperature treatments, respectively. Given such stability in temperature across triplicate aquaria at both control (1-way ANOVA effect of tank, df = 2, F = 0.719, p = 0.490) and experimental (df = 2, F = 0.384, p = 0.680) temperatures, pseudo-replicated nubbins (n = 3 per aquarium) were pooled across aquaria for each treatment, resulting in nine biological replicates for each time and treatment and 90 RNA/DNA/protein extractions.

### Genome copy proportions (GCPs)

**Figure 4:**
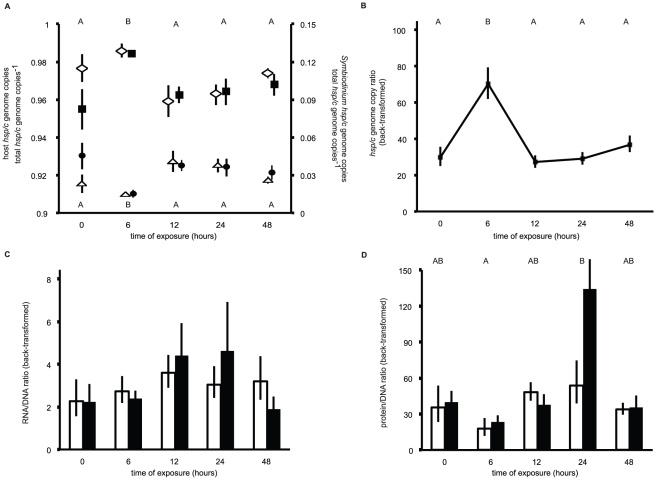
Genome copy proportions (GCP), genome copy ratio (GCR), RNA/DNA and protein/DNA ratios from the thermal stress experiment. (A) Host (left y-axis) and *Symbiodinium* (right y-axis) *hsp/c* genome copies as a proportion of the total *hsp/c* genome copies within the sample (GCP). Hollow diamonds and triangles represent data from the control treatment for the host and *Symbiodinium* GCPs, respectively. Filled squares and circles represent data from the high temperature treatment for the host and *Symbiodinium* GCPs, respectively. Tukey's HSD letter groups (α<0.05) above and below icons correspond to the host and *Symbiodinium* data, respectively, and test only temporal variation, as neither significant treatment nor interaction effects were observed for these parameters. While both host and *Symbiodinium* GCP appear to differ between treatments at time = 0, this difference was not statistically significant (Tukey's post hoc test, p>0.05). (B) Mean host/*Symbiodinium hsp/c* GCRs pooled across both treatments over time. Tukey's HSD letter groups (α<0.05) are also presented. (C) RNA/DNA ratio over time for both control (white columns) and high (black columns) temperature treatments. (D) Protein/DNA ratio over time for both control (white columns) and high (black columns) temperature treatments. Tukey's HSD letter groups test only temporal variation, as neither significant treatment nor interaction effects were observed for this parameter. While the protein/DNA ratio appears to differ between treatments at time = 24, this difference was not statistically significant (Tukey's post hoc test, p>0.05). In all panels, error bars represent standard error of the mean, and for panels B–D, the back-transformed means are presented.

Host GCP data were rank-transformed prior to a 2-way ANOVA on ranks [33], while the *Symbiodinium* GCPs were log-transformed prior to a standard 2-way ANOVA. Neither host nor *Symbiodinium hsp/c* GCPs (Fig. 4A) varied significantly between temperatures (Table 2). However, they did change significantly over time, primarily due to a significant drop in the *Symbiodinium* GCP, and consequent increase in the host GCP, in both treatments after 6 hours of exposure (see Tukey's [temporal only] HSD groups of Fig. 4A). Given the statistically similar GCP values for both host and *Symbiodinium* at each of the sampling times, the data were pooled across treatments at each time, and a host/*Symbiodinium* genome copy ratio (GCR) was calculated and plotted (Fig. 4B) to further depict the change in the composition of the coral holobiont over the duration of the experiment. Accordingly, the GCR also changed significantly over time (1-way ANOVA effect of time, df = 4, F = 9.626, p<0.001), predominantly due to a 2.5-fold increase in samples collected at 6 hours relative to the other sampling times (see Tukey's HSD groups of Fig. 4B).

**Table 2:** 2-way ANOVA table for molecular data from the thermal stress experiment.

Source of variation	df	MS	F	p
***S. hystrix hsp/c*** genome copy proportion (DNA fraction)*				
Temperature	1	487	1.414	0.239
Time	4	3038	8.821	**0.000**
Temperature×Time	4	212	0.617	0.652
***Symbiodinium hsp/c*** genome copy proportion (DNA fraction)**				
Temperature	1	0.041	0.857	0.358
Time	4	0.424	8.917	**0.000**
Temperature×Time	4	0.021	0.433	0.784
**Host/** ***Symbiodinium hsp/c*** genome copy ratio (DNA fraction)**				
Temperature	1	0.044	0.876	0.353
Time	4	0.447	8.829	**0.000**
Temperature×Time	4	0.022	0.442	0.778
***S. hystrix hsp/c*** expression (RNA fraction)**				
Temperature	1	0.008	0.071	0.791
Time	4	0.972	8.924	**0.000**
Temperature×Time	4	0.017	0.152	0.961
***Symbiodinium hsp/c*** gene expression (RNA fraction)**				
Temperature	1	0.277	2.875	0.095
Time	4	1.727	17.915	**0.000**
Temperature×Time	4	0.103	1.065	0.381
Total holobiont soluble protein polyp^−1^ (protein fraction)**				
Temperature	1	2.2×10^−6^	1×10^−4^	0.994
Time	4	0.188	1.183	0.334
Temperature×Time	4	0.039	0.247	0.910
RNA/DNA ratio**				
Temperature	1	0.204	0.026	0.872
Time	4	12.497	1.578	0.194
Temperature×Time	4	6.410	0.826	0.515
Protein/DNA ratio**				
Temperature	1	0.050	0.621	0.438
Time	4	0.257	3.205	**0.029**
Temperature×Time	4	0.042	0.523	0.720

Data were either rank (“*”) or log (“**”) transformed, and statistically significant differences are highlighted in bold.

### RNA/DNA and protein/DNA ratios

Neither the RNA/DNA (Fig. 4C) nor the protein/DNA (Fig. 4D) ratio varied in response to temperature (Table 2), though the latter changed significantly over time, with lowest levels (average of both treatments  = ∼15) measured after 6 hours of exposure and highest levels (∼90) measured at 24 hours (see Tukey's HSD [temporal only] groups of Fig. 4D). The RNA/DNA ratio was approximately 3 for the control samples (pooled across all times) and 3.4 for the high temperature samples, though this difference was not statistically significant. Likewise, the overall average protein/DNA ratio for the control samples was 38, approximately 1.4-fold lower than that of the high temperature treatment samples (∼54); however, this difference was not statistically significant.

### Molecular chaperone gene expression

**Figure 5:**
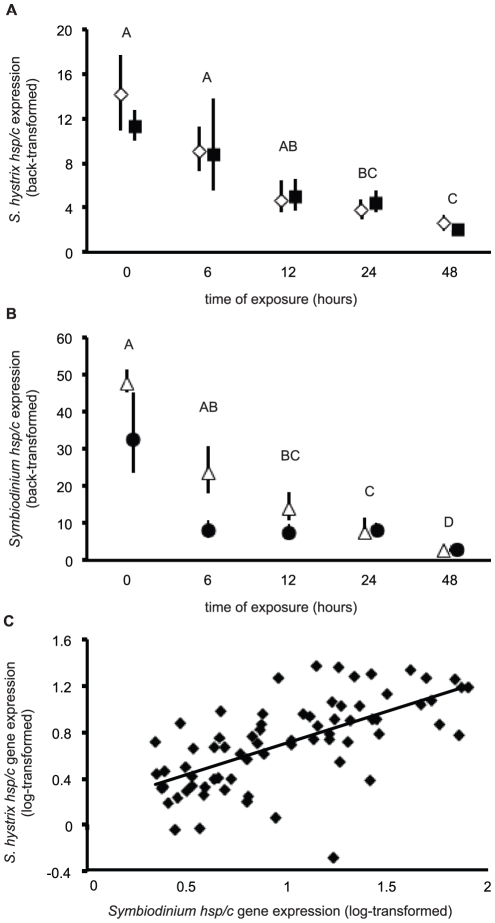
Host and *Symbiodinium hsp/c* mRNA expression results from the thermal stress experiment. (A) Diamonds and squares represent mean host coral *hsp/c* expression for the control and elevated temperature treatments, respectively. (B) Triangles and circles represent mean *Symbiodinium hsp/c* expression for the control and elevated temperature treatments, respectively. In both panels, error bars represent standard error of the back-transformed mean, and Tukey's post-hoc groups represent α<0.05 significance levels for temporal variation only, as there were no significant treatment or interaction effects on host or *Symbiodinium hsp/c* gene expression (Table 2). In some cases, error bars do not extend beyond the icon. (C) The correlation between host and *Symbiodinium* log-transformed *hsp/c* expression across all samples.

Both host and *Symbiodinium hsp/c* gene expression data were log-transformed to achieve the normal distribution required for ANOVA. *S. hystrix hsp/c* expression (Fig. 5A) was not induced by exposure to 30°C (Table 2), though it did change significantly over time (see Tukey's [temporal only] HSD groups of Fig. 5A). The highest levels of expression were measured at the initial sampling time (t = 0), and in both control and experimental colonies, the expression decreased ∼4-fold between t = 0 and t = 48 hours.

Similarly, *Symbiodinium hsp/c* expression (Fig. 5B) was not significantly affected by temperature, though it did change significantly over time (Table 2). The highest expression was measured in samples collected at t = 0, while lowest expression was found in both control and experimental colonies sampled after 48 hours (see Tukey's HSD groups in Fig. 5B), and there was an approximately 10-fold decrease in expression over the duration of the experiment. Finally, there was a statistically significant correlation (Fig. 5C) between host and *Symbiodinium hsp/c* gene expression within a biopsy across all samples (r^2^ = 0.402, linear regression t-test, t = 6.940, p<0.001). Statistically significant correlations in expression of the *hsp/c* gene between coral and *Symbiodinium* were also observed amongst both control (r^2^ = 0.375, t = 4.690, p<0.001) and experimental (r^2^ = 0.431, t = 5.180, p<0.001) samples.

### Total holobiont soluble protein (THSP)

**Figure 6:**
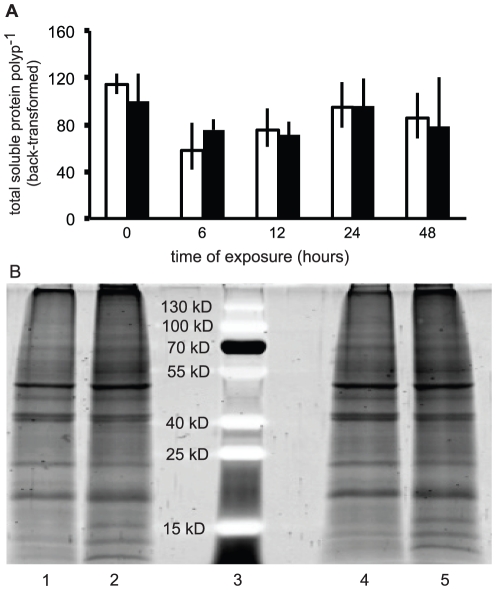
Total holobiont soluble protein per polyp and a representative SDS-PAGE gel from the thermal stress experiment. (A) Proteins were extracted from the same samples from which RNA and DNA were isolated, and error bars represent standard error of the back-transformed mean for both control (white columns) and experimental (black columns) samples. (B) A representative SDS-PAGE gel (20 µg protein) from two randomly chosen samples from both the control (lanes 1–2, representing samples from time = 0 and 6, respectively) and elevated temperature (lanes 4–5, representing samples from t = 0 and 6, respectively) treatments. A pre-stained protein ladder (lane 3) is also depicted.

Protein co-extracted from the same biopsies from which RNA and DNA were isolated (Fig. 6A) proved to be of high quantity (mean yield = 91 +/40 [standard deviation of the mean] µg polyp^−1^) and quality (Fig. 6B). However, there was no significant effect of temperature, time, or their interaction on (log-transformed) THSP/polyp (Table 2). The protein/DNA ratio (see above), however, was significantly temporally variable.

## Discussion

The cDNA library generated herein was not normalized prior to cloning, meaning that a notable degree of redundancy was observed for some genes. For instance, 54 clones were assembled into only two CCAAT enhancer-binding protein contigs (Table S1). Also, it appears that the poly-A selection was not entirely effective, as a significant number of not only poly-A tail-lacking bacterial sequences, but also 35 rRNAs, were identified. While these two characteristics did not allow for the maximum degree of gene discovery, they did generate a more accurate snapshot of the composition of the holobiont from an RNA perspective.

Of the 804 high quality, full length, non-bacterial cDNA clones, 73.5% were of presumed anthozoan origin, 8.5% from *Symbiodinium*, and 18% from either coral or *Symbiodinium* (“holobiont”). If the assumption is made that the host represents a similar proportion of the holobiont fraction as it does in the identified fraction (73.5%/[73.5%+8.5%] or 89.7%), then the total clone breakdown would be ∼90% coral and ∼10% *Symbiodinium*. This agrees fairly well with the genome copy proportions and ratios (Fig. 4), which found that approximately 95% of the total *hsp/c* genome copies were from the coral. However, this DNA proxy may not necessarily reflect the biomass ratio of host/*Symbiodinium* within each sample, as it is likely that each member possesses a different number of *hsp/c* genome copies per cell. Given the absence of a fully sequenced genome for either member, the GCP should only be used to calculate the relative *changes* in host and *Symbiodinium* biomass between samples, not to accurately convey the host/*Symbiodinium* biomass ratio within an individual sample; only the former calculation is necessary to appropriately normalize gene and protein expression data, as was conducted herein. It seems probable, then, that the proportional breakdown of sequenced cDNAs would represent a better approximation for the biomass ratio of this coral. As such, *S. hystrix* appears to be approximately 90% animal and 10% dinoflagellate, though this ratio was not stable over time.

Due to the short-term nature of this experiment, bleaching was not anticipated, and thus it was hypothesized that the host and *Symbiodinium hsp/c* GCPs would be unresponsive to treatment. While this was indeed the case, there was a statistically significant decrease in *Symbiodinium* GCP after 6 hours, as well as a statistically significant increase in host GCP, in corals of both the control and experimental treatments, suggesting expulsion of *Symbiodinium* or host cell division. As elevated *in hospite Symbiodinium* densities have been documented in this coral at night [28], the former scenario seems more likely. However, the high rates of host protein turnover suggested by the *hsp/c* data, described below, may indicate elevated levels of host cell turnover, supporting the latter hypothesis. Given that mitotic indices of *Symbiodinium* within this coral species have previously been found to be quite low (1–2% [9]), the significantly higher division rates deduced from the number of genome copies quantified suggests that the biological make-up of this coral species may be more temporally dynamic than was previously thought. Ultimately, though, the fact that the biological composition of the coral holobiont significantly changed over a 48 hour experiment in even the control samples necessitates that *S. hystrix* gene and protein expression data are normalized to account for such variation.

Before presenting several hypotheses as to why the hypothesized *hsp/c* induction was not observed, it is important to mention the previous body of work on the *hsp70* gene/protein in corals (e.g., [13]–[25]). Indeed, many studies (e.g., [16]) have found *hsp70* mRNA or protein expression to be induced in specimens exposed to elevated temperatures. However, these authors neglected to consider the endosymbiotic nature of corals in their experiments, when actually, it is imperative to consider the fact that a healthy reef coral is composed of both anthozoan and protozoan components [31]. For instance, a bleached coral is composed of a greater proportion of animal material relative to a healthy coral, and unless appropriate controls are taken, researchers will measure and report higher levels of coral gene or protein expression in the former, and similarly, higher levels of *Symbiodinium* gene or protein expression in the latter. Furthermore, housekeeping genes common in model organisms, such as β*-actin*, have been shown to undergo diel fluctuations in expression in corals [28], invalidating their utility in studies with certain reef corals. Therefore, while a sizable body of work on the *hsp70* gene/protein exists for corals, it is not possible to compare these results with our own. Instead, researchers are urged to re-assess the validity of the *hsp70* gene/protein as a temperature-responsive molecule after normalizing their data within a framework that accounts for the dual-compartmental nature of corals.

There are several potential reasons why, in general, *hsp/c* expression decreased over time in both endosymbiotic compartments yet did not differ between treatments in either. First, the quantity of mRNA inside a cell is a function of both *de novo* transcription and the half-life of pre-existing transcripts, and it has been shown that *hsp70* mRNA is more stable under stressful conditions as a strategy to obtain more protein translation from the same mRNA [34]. However, when the organism is continuously subjected to stressful conditions, the half-life of the mRNA decreases [35]. Given the short-term nature of these experiments, the lack of induction may be suggestive of this elevated *hsp70* mRNA half-life phenomenon. However, biogeography may also have played a role in the absence of a molecular chaperone response.

While ephemeral exposure to 30°C was previously shown to be detrimental to the health of *S. hystrix* colonies [9]–[10], [27], specimens used herein may not have produced increased levels of *hsp/c* mRNA transcripts when exposed to elevated temperatures due to having routinely been subjected to highly variable temperatures in the reef environment from which they originated (Fig. 3). Nanwan Bay experiences seasonal upwelling [36], and, as such, many corals experience temperature fluctuations from 23–30°C on a given day [37], potentially giving them a remarkable degree of plasticity in response to dramatic temperature changes [38]. Therefore, constitutively expressing high levels of molecular chaperone mRNAs may be seen as a defense mechanism that enables them to quickly translate large quantities of the respective protein in response to rapid temperature changes that occur during upwelling events. In fact, this is a common strategy in intertidal invertebrates (e.g., [39]), and has been suggested to be important in corals, as well [40]. This idea is currently being explored by reciprocal transplant studies in which *S. hystrix* colonies from stable (25–29°C over the year) water temperature environments are transported to Nanwan Bay and vice versa. Interestingly, though, a similar coral reciprocal transplant study [21] failed to observe pronounced HSP70 induction in response to differential thermal regimes.

In addition to, or in place of, the aforementioned hypotheses, it is also possible that, while HSP70 likely functions in rectification of coral and *Symbiodinium* protein damage, its constitutive role in regulating protein turnover may essentially mask the ability to detect induction under periods of thermal stress. In other words, due to high rates of protein turnover caused by, perhaps, *Symbiodinium* cell turnover (Fig. 4 and [28]), HSP70, and hence *hsp/c*, may be constitutively expressed at high levels. Therefore, given the intermediate homology of the isolated host and *Symbiodinium hsp/c* orthologs between inducible and constitutive isoforms, it is possible that these genes actually only encode the latter, thus explaining the relatively high levels of *hsp/c* expression in both hosts and *Symbiodinium* from the control colonies (Fig. 5A–B).

Despite the lack of *hsp/c* induction in either member of the *S. hystrix-Symbiodinium* endosymbiosis, there was a significant correlation in mRNA expression within samples across all samples of both treatments (Fig. 5C). This significant correlation of the same ortholog within the coral holobiont across treatments further emphasizes the notion that considering only one member of the endosymbiosis in molecular analyses will not only preclude the ability to accurately measure gene or protein expression, but it may also lead researchers to overlook critical insight into the molecular regulation of these endosymbioses and how their utilization of conserved genes and proteins differs in response to environmental stress. For instance, it appears in this case that the correlation in expression of the *hsp/c* molecular chaperone across two eukaryotes engaged in an endosymbiosis may be more informative of their physiological response to elevated temperatures than the absolute expression levels of this gene. As such, while the function of the *hsp/c* ortholog could not be deduced solely based on *in silico* or gene expression analyses, the statistically significant correlation in expression potentially suggests that the isolated orthologs play a similar role in each member of this coral endosymbiosis. This role, however, appears to preclude the ability of this molecular chaperone paralog to be a useful, mRNA-level molecular biomarker for assessing pre-bleaching levels of thermal stress in either member of this endosymbiosis. Therefore, future work will seek to validate alternative temperature-sensitive mRNAs from the cDNA library developed herein, and, in fact, a series of qPCR assays have already been designed for such purposes (Table S2).

Of the three macromolecular fractions; RNA, DNA, and protein, extracted from each of the 90 coral biopsies, the latter (Fig. 6) showed the least significant change over time and between treatments (Table 2). In contrast, both gene expression (RNA fraction) and GCPs (DNA fraction) demonstrated significant temporal variation (Table 2). While it is tempting to conclude that THSP/polyp is an uninformative analyte, it should be noted that, unlike the RNA and DNA parameters, THSP/polyp was not normalized in an endosymbiosis-specific context. For instance, corals sampled at 6 hours, which were shown to have a relatively elevated level of host biomass relative to the total biomass of the holobiont (Fig. 4), likely had a protein composition that reflected this state. This is potentially revealed in the protein/DNA ratio (Fig. 4D), which, unlike THSP, varied significantly over time and was at lowest levels after 6 hours. Given that this coral was shown to be approximately 10% dinoflagellate, the drop in *Symbiodinium* density at 6 hours could have led to this simultaneous decrease in protein content/DNA. Thus, it is recommended that if researchers seek to analyze expression of specific proteins, they should normalize their data in an analogous manner as was conducted herein for gene expression in order to gain resolution and minimize bias associated with analyzing macromolecules isolated from endosymbiotic corals, whose composition with respect to RNA, DNA and protein appears to be temporally variable.

This study highlights the notion that, in order to create a more composite picture of the sub-cellular response of reef corals and their endosymbiotic dinoflagellate communities to environmentally relevant increases in temperature, it is fruitful to take a multi-macromolecule approach in order to assess a variety of parameters across RNA, DNA, and protein fractions. Herein, temporal variation was shown to be a more important determinant in both molecular chaperone expression and biological composition of the coral holobionts. This observation highlights the need to not only assay a variety of analytes across biological scales, but also suggests that studies focusing only on terminal time points, and not those intervening, may miss moments of critical physiological changes driven by factors such as the light cycle or an intrinsic circadian rhythm. Finally, this work emphasizes the potential importance of assessing gene expression data across compartments (i.e., within biopsies), providing a useful conceptual platform for other researchers interested in determining how biological composition of an endosymbiosis influences the gene-level response of its constituents to environmental change.

## Materials and Methods

### cDNA library generation

Small (2 cm) branches were removed *in situ* from five randomly selected *S. hystrix* colonies collected on SCUBA at Houbihu (21°56′18.01″N, 120°44′45.54 E), a small embayment within Nanwan Bay, Taiwan's southernmost bay. Prior to collection, a permit (KNP: 0992900398) was obtained from Kenting National Park. These fragments were frozen in liquid nitrogen, partially homogenized with a mortar and pestle, and then further homogenized in 1 ml TRIzol® (Invitrogen). RNA was extracted following the manufacturer's recommendations, and RNAs from the five samples were pooled, further purified according to the manufacturer's recommendations with the Fast Track® mRNA isolation kit (Invitrogen) to remove rRNA, tRNA and residual DNA, eluted into DECP-treated water and assessed for quantity and quality on a Nanodrop spectrophotometer (Infinigen) and 0.8% formaldehyde-agarose gels stained with ethidium bromide, respectively. mRNA (3 µg) was converted to cDNA and cloned *sensu* [41], and 1,092 clones were sequenced with the T7 primer on a Prism 377 DNA analyzer (Applied Biosystems) with BigDye® terminator cycle sequencing chemistry (Applied Biosystems, manufacturer's recommendations).

After sequencing from the 5′ end, clones possessing inserts >100 bp in which >99% of the bases could be called accurately were trimmed of residual vector sequence and checked for the presence of a poly-A tail *sensu* [42]. If such was not found, another sequencing primer unique to the clone of interest was designed from the initial read and used to re-sequence the clone to completion. An initial translating BLAST (tBLASTx) of the ensuing sequences was used to eliminate clones representing *E. coli* or other culture contaminants (Fig. 1). Then, tBLASTx was conducted to assign identity to clones, or, in some cases, assembled contigs, using a stringency threshold of e<10^−6^. Clones in which the top BLAST hit was an animal were assumed to be of coral origin. Likewise, clones most closely resembling published protozoan and bacterial sequences were assumed to be of *Symbiodinium* and bacterial origin, respectively. Gene ontologies of the clones assigned to either coral or *Symbiodinium* were ascribed with BLAST2Go [43] and KAAS [44], and these clones were then categorized into functional groups. If the top BLAST hit had not been functionally characterized, the respective clone was assigned to the “unknown function” category.

Clones which did not pass the e<10^−6^ threshold were subsequently BLASTed (tBLASTx) against “PdamBase” (http://cnidarians.bu.edu/PdamBase, [29]), which contains sequences from the entire coral “holobiont” (host, *Symbiodinium*, and other associated microbes). Clones significantly (e<10^−6^) aligning to sequences in this database were deemed “holobiont” because their origin could not be confidently attributed to one particular member of the holobiont.

### Field data

Temperature was measured *in situ* at Houbihu with HOBO temperature loggers at 60 min intervals for 6 months prior to experimental work in order to choose suitable acclimation and control temperatures for the temperature manipulation experiment. Logging of seawater temperature continued until a full year's worth of data had been accumulated.

### Thermal stress experiment

*S. hystrix* is a temperature-sensitive species and will readily bleach upon ephemeral (several days) exposure to temperatures greater than 0.5–1°C above the average annual maximum [45]. As the maximum monthly mean temperature of Houbihu is approximately 29°C (September, Fig. 3), it was hypothesized that 30°C would be a sufficiently elevated temperature to observe a physiological response in *S. hystrix* [27]. Therefore, in June 2009, 18 *S. hystrix* colonies (1,500–2,000 cm^3^) were collected from Houbihu, randomly assigned to 1 of 6 aquaria (300 l, 3 pseudo-replicated colonies per aquarium) and acclimated for 3 weeks at 27°C (the ambient temperature at the time of collection, Fig. 3) and 300–400 µeinsteins m^−2^ s^−1^ on a 13 hr∶11 hr (07:00–20:00) light∶dark cycle at Taiwan's National Museum of Marine Biology and Aquarium (NMMBA). Afterwards, 50 mg samples were removed from each colony and submerged in 500 µL TRI-Reagent® (Ambion) to serve as time = 0 (0:700) samples. The number of polyps from each sample was recorded and used for normalization of protein data.

Three of the six aquaria were randomly chosen to serve as the experimental treatment, and the temperature was ramped to 30°C over 3 hours. Given the interest of this study in the pre-bleaching stress response of *S. hystrix* and its *in hospite Symbiodinium* populations, the experiment was conducted over the course of only 48 hours. Sampling was performed 6 (13:00), 12 (19:00), 24 (0:700), and 48 (0:700) hours after initial temperature ramping, resulting in the production of 90 samples that were immediately immersed in TRI-Reagent and frozen at −80°C prior to extraction. As the same 18 coral colonies were sampled five times, the assumption was made that sampling of a portion of the colony representing less than 0.1% of the total colony volume would not cause stress to another section of that same colony. Finally, as artificial lighting was used during the experiment, all samples were taken while at the same light level.

### RNA/DNA extractions and cDNA synthesis

Coral fragments frozen in 500 µl TRI-Reagent were thawed, and an additional 500 µl TRI-Reagent spiked with 10 pg somatolactin (*sl*) RNA was added. RNA and DNA were extracted *sensu* [31] except that 2 µl Co-Precipitant Pink™ (Bioline), 0.1 vol sodium acetate (3 M, pH 5.2) and 1 vol isopropanol were used for DNA precipitation. RNA (10 µl) was treated with RQ1 RNase-free DNase (Promega, manufacturer's recommendations). However, in place of heat denaturing the enzyme, which previously led to RNA degradation due to strand scission from divalent cations found in the DNase buffer (data not shown), 1 ml TRI-Reagent was added to the RNA, which was then re-extracted. RNA and DNA quantity and quality were assessed as above, and an RNA/DNA ratio was calculated for each sample as a proxy for total gene transcription. RNA (10 µl) was reverse-transcribed with a kit (“High Capacity™,” Applied Biosystems) following the manufacturer's recommendations.

### Real-time quantitative PCR

*Symbiodinium* primers for an *hsp70*-like gene [31], deemed “*hsp/c*” due to its intermediate homology between inducible (HSP) and constitutive (HSC) isoforms, were utilized for triplicate qPCRs of both DNA and cDNA following the author's recommendations. Host coral primers (forward: 5′-AATCCAGGAGTTGCTCAGAAATT-3′ and reverse: 5′-TCTGACACAGCTTCACTCTTA-3′) were designed (Fig. 2B) from a cDNA clone (NCBI accession: HM147130) whose respective protein's homology was also intermediate between HSP and HSC isoforms (Fig. 2A) and was likewise deemed *hsp/c*. These primers amplified a 135 bp fragment from both DNA and cDNA. In a 20 µl reaction, 500 nM each primer, 100 ng BSA, and 1× Power® SYBR Green mastermix (Applied Biosystems) were mixed with 2 µl undiluted cDNA or 2 µl DNA (diluted to 10 ng µl^−1^). After a 10 min incubation at 95°C, qPCRs were carried out over 35 cycles of 95°C for 15 s and 59°C for 60 s in an Applied Biosystems 7500 real-time PCR machine. A melt curve was conducted after each run for both host and *Symbiodinium hsp/c* assays to ensure that primers were specific to the ortholog and organism of interest, a concern given the strong homology between *hsp* and *hsc* genes of both the coral and *Symbiodinium* (Fig. 2B; [46]). PCR efficiency of each primer set was estimated by amplifying serially diluted cDNAs or DNAs and calculating the slope of the best-fit line [47].

### Gene expression data analysis

After calculating *hsp/c* genome copy (DNA fraction) threshold cycles (C_t_) for both host and *Symbiodinium*, a proportion of the total (both host and *Symbiodinium*) *hsp/c* genome copies was computed for each member as follows to compute the genome copy proportion (GCP), or, in other words, the proportion of the total genome copies attributed to each of the two dominant eukaryotes of this coral-dinoflagellate endosymbiosis:

(E_Sym *hsp/*c_^Ct^_Sym hsp/c_)^−1^/((E_Sym *hsp/c*_^Ct^_Sym hsp/c_)^−1^+(E_host *hsp/c*_^Ct^_host *hsp/c*_)^−1^) for the *Symbiodinium* GCP, and (E_host *hsp/c*_^Ct^_Sym *hsp/c*_)^−1^/((E_Sym *hsp/c*_^Ct^_Sym *hsp/c*_)^−1^+(E_host *hsp/c*_^Ct^_host *hsp/c*_)^−1^) for the host coral GCP. In both equations, “E” refers to the efficiency of the respective qPCR primer set and is taken to the power of C_t_. This GCP was used to normalize gene expression, as prior work has shown that the composition of the coral-dinoflagellate endosymbiosis can bias gene expression data [31]. For instance, a partially bleached coral will have a greater proportion of host RNA relative to the total RNA of the holobiont, and this must be considered in gene expression analyses, lest higher levels of host gene expression be inappropriately attributed to the bleached samples. While bleaching was not anticipated in this study, natural variation in *Symbiodinium* densities also stands to bias gene expression data, and such has indeed been documented in this coral [28].

For the cDNA-derived data, host and *Symbiodinium hsp/c* expression were normalized to the *sl* RNA spike *sensu* [31] to control for reverse transcription differences between samples. A previous study on *S. hystrix* found that commonly utilized housekeeping genes for relative quantification of gene expression, such as *B-actin* and *a-tubulin*, actually undergo diel fluctuations in expression [28], emphasizing that exogenous RNA spikes are a more appropriate gene expression normalization strategy for this coral. Then, spike-normalized host *hsp/c* gene expression was divided by the host GCP. *Symbiodinium* spike-normalized *hsp/c* gene expression was analogously normalized to the *Symbiodinium hsp/c* GCP. This DNA-based normalization controls for potentially different quantities (and/or proportions) of host or *Symbiodinium* nucleic acids between samples [31]. Finally, the *sl* spike and GCP-normalized *hsp/c* expression for both host and *Symbiodinium* were normalized to total RNA (µg) to control for biopsy biomass differences. Two-way ANOVAs were performed with JMP (version 5.0) to assess the effects of temperature, time, and their interaction on both the host and *Symbiodinium* GCP and normalized *hsp/c* gene expression after checking for normality and homogeneity of variance of the data. JMP was then used to test the statistical significance of the correlation between log-transformed host and *Symbiodinium hsp/c* gene expression across all samples. In all cases, an α<0.05 was established.

### Protein extraction and SDS-PAGE

After removal of the aqueous DNA phase, proteins were extracted from the same samples as for RNA and DNA according to the recommendations provided by Ambion. Briefly, proteins were precipitated with acetone at −20°C for 1–2 hours, washed thrice at room temperature (RT) with a solution of 0.3 M guanidine HCl in 95% ethanol (supplemented with 2.5% glycerol), washed once at RT with a 95% ethanol/2.5% glycerol solution, and dried on the benchtop for 5–10 min. During RT incubations in the wash solutions, proteins were sonicated to completely disassociate aggregates. After drying, proteins were re-suspended in 1× “sample” buffer [48], boiled at 100°C for 5 min, and spun at 12,000× g for 10 min at 4°C. The protein concentration of the supernatant was quantified with the 2-D Quant™ kit (Amersham Biosciences) according to the manufacturer's recommendations, and a protein/DNA ratio was calculated for each sample in order to generate a proxy for total protein translation.

Approximately 20 µg protein was electrophoresed through 4% stacking and 12% separating SDS-PAGE gels at 75 V for 30 min and 130 V for 90 min, respectively, using the Bio-Rad Mini-PROTEAN™ Tetrad system. Gels were stained with SYPRO® Ruby (Bio-Rad) according to the manufacturer's recommendations to visualize protein quality. Total holobiont soluble protein (THSP) levels from samples exhibiting high quality protein on SDS-PAGE gels were compared across time and treatments in a 2-way ANOVA, as above, after normalizing to number of polyps used in the initial RNA/DNA/protein extractions. Western blotting with a commercially available HSP70 monoclonal antibody (Stressgen, Cat. #822) was performed *sensu* [21], though due to the inability to recognize this protein in the majority of samples (only about 10% of samples produced a visible band [data not shown]), these data were not included herein.

## Supporting Information

Table S1 **Selected** ***Seriatopora hystrix*** **clones spanning 19 functional categories.** When multiple clones encoding the same protein were identified, only a representative sequence was chosen for publication on the NCBI database. Real-time quantitative PCR assays (Table S2) were designed for use in future studies for genes noted by an “*.” Genes characterized in [27] are denoted by an “^a^.” The *hsp/c* gene of interest in this study is highlighted in bold italics.
(DOCX)

Table S2 **Real-time quantitative PCR primers designed for use with SYBR® green I chemistry with whole holobiont total RNA extracts from** ***Seriatopora hystrix*** **.** Assays in which it was necessary to dilute the cDNA 10-fold in order to achieve optimal results are denoted by “^a^.” For all assays, each cycle included a 95°C hold for 15 s, followed by a 60 s incubation at the respective annealing temperature.
(DOCX)
