# Supplementary material for: Assessing the Impacts of Experimentally Elevated Temperature on the Biological Composition and Molecular Chaperone Gene Expression of a Reef Coral
Source: PLoS One. 2011 Oct 27;6(10):e26529. doi: 10.1371/journal.pone.0026529 (PMC3203140; doi:10.1371/journal.pone.0026529)
Supplement: Table S1 — Selected Seriatopora hystrix clones spanning 19 functional categories. When multiple clones encoding the same protein were identified, only a representative sequence was chosen for publication on the NCBI database. Real-time quantitative PCR assays (Table S2) were designed for use in future studies for genes noted by an “*.” Genes characterized in [27] are denoted by an “a.” The hsp/c gene of interest in this study is highlighted in bold italics. (DOCX) [file pone.0026529.s001.docx]

| ***Seriatopora hystrix* gene NCBI accession Top tBLASTx hit (species) e value clones (#)** | | | | |
| --- | --- | --- | --- | --- |
| **CALCIUM REGULATION** | | | | |
| calcium and integrin-binding family protein | JN244387 | *Nematostella vectensis* | 10^-108^ | 2 |
| calcium transporting ATPase | JN244389 | *Strongylocentrotus purpuratus* | 10^-52^ | 1 |
| calcium-binding EGF-like domain | JN244388 | *Nematostella vectensis* | 10^-38^ | 1 |
| extracellular calcium binding domain | JN244390 | *Nematostella vectensis* | 10^-37^ | 1 |
| **CELL DIVISION** | | | | |
| transforming growth factor β | JN244396 | *Heliocidaris erythrogramma* | 10^-11^ | 3 |
| septin 7 | JN244393 | *Nematostella vectensis* | 10^-169^ | 1 |
| radial spoke head 1 | JN244394 | *Nematostella vectensis* | 10^-108^ | 1 |
| chromosome segregation protein SMC | JN244391 | *Strongylocentrotus purpuratus* | 10^-40^ | 1 |
| PAX interacting protein 1 | JN244395 | *Nematostella vectensis* | 10^-15^ | 1 |
| **CYTOSKELETON** | | | | |
| β-actin^a^ | HM147127 | *Seriatopora hystrix* | 0 | 6 |
| α-tubulin^a^ | HM147129 | *Seriatopora hystrix* | 0 | 2 |
| myosin light chain | JN244397-98 | *Nematostella vectensis* | 10^-74^ | 2 |
| β-tubulin | JN244403 | *Halocynthia roretzi* | 0 | 1 |
| ezrin* | HM147133 | *Seriatopora hystrix* | 0 | 1 |
| tropomyosin^a^ | HM147128 | *Seriatopora hystrix* | 0 | 1 |
| filamin-A | JN244400 | *Nematostella vectensis* | 10^-77^ | 1 |
| myosin heavy chain | JN244399 | *Mytilus galloprovincialis* | 10^-34^ | 1 |
| dystrophin | JN244402 | *Nematostella vectensis* | 10^-16^ | 1 |
| cofilin | JN244404 | *Nematostella vectensis* | 10^-14^ | 1 |
| **DEVELOPMENT** | | | | |
| yolk protein | JN244412 | *Saccoglossus kowalevskii* | 10^-33^ | 3 |
| neural proliferation differentiation protein 1 | JN244405 | *Nematostella vectensis* | 10^-58^ | 2 |
| zona pellucida-like domain | JN244413 | *Nematostella vectensis* | 10^-50^ | 2 |
| vitellogenin | JN244411 | *Saccoglossus kowalevskii* | 10^-33^ | 2 |
| dickkopf-related protein 3 | JN244410 | *Nematostella vectensis* | 10^-81^ | 1 |
| chaperone for wingless signaling and trafficking  of LDL receptor | JN244406 | *Nematostella vectensis* | 10^-38^ | 1 |
| segmentation protein capncollar | JN244408 | *Nematostella vectensis* | 10^-29^ | 1 |
| DM domain protein | JN244409 | *Acropora millepora* | 10^-27^ | 1 |
| egg protein | JN244407 | *Galaxea fascicularis* | 10^-24^ | 1 |
| **DNA STRUCTURE AND REPAIR** | | | | |
| methyl-CpG-binding domain protein | JN244418 | *Nematostella vectensis* | 10^-62^ | 2 |
| DNA excision repair protein | JN244415 | *Strongylocentrotus purpuratus* | 10^-78^ | 1 |
| poly (ADP-ribose) polymerase | JN244414 | *Canis familiaris* | 10^-54^ | 1 |
| bromo adjacent homology domain protein | JN244419 | *Nematostella vectensis* | 10^-45^ | 1 |
| chromodomain DNA helicase binding protein 1 | JN244420 | *Danio rerio* | 10^-40^ | 1 |
| cryptochrome 2 photolyase | JN244417 | *Nematostella vectensis* | 10^-40^ | 1 |
| nucleosome assembly protein 1 | JN244422 | *Nematostella vectensis* | 10^-28^ | 1 |

**Table S1**. **Selected *Seriatopora hystrix* clones spanning 19 functional categories**. When multiple clones encoding the same protein were identified, only a representative sequence was chosen for publication on the NCBI database. Real-time quantitative PCR assays were designed for use in future studies for genes noted by an “*.” Genes characterized in [28] are denoted by an “^a^.” The *hsp/c* gene of interest in this study is highlighted in bold italics.

| ***Seriatopora hystrix* gene NCBI accession Top tBLASTx hit (species) e value clones (#)** | | | | |
| --- | --- | --- | --- | --- |
| **EXTRACELLULAR INTERACTIONS** | | | | |
| usherin | JN244430 | *Nematostella vectensis* | 10^-167^ | 1 |
| xotch protein | JN244424 | *Nematostella vectensis* | 10^-112^ | 1 |
| echinonectin | JN244429 | *Strongylocentrotus purpuratus* | 10^-75^ | 1 |
| matrix metallopeptidase | JN244427 | *Nematostella vectensis* | 10^-29^ | 1 |
| periostin precursor | JN244428 | *Salmo salar* | 10^-29^ | 1 |
| hemicentrin 1 | JN244425 | *Danio rerio* | 10^-23^ | 1 |
| fibrillin 1 | JN244426 | *Ciona intestinalis* | 10^-12^ | 1 |
| **LIPID METABOLISM AND TRAFFICKING** | | | | |
| phospholipase α2* | HM156700 | *Anolis carolinensis* | 10^-48^ | 3 |
| phosphaditylinositol transfer protein | JN244432 | *Nematostella vectensis* | 10^-17^ | 2 |
| apolipoprotein L3 | JN244435 | *Pocillopora damicornis* | 10^-130^ | 1 |
| phosphaditylserine synthase | JN244438 | *Pocillopora damicornis* | 10^-54^ | 1 |
| pancreatic lipase | JN244437 | *Mizuhopecten yessoensis* | 10^-30^ | 1 |
| triacylglycerol lipase | JN244436 | *Nematostella vectensis* | 10^-29^ | 1 |
| **METABOLISM** | | | | |
| carbonic anhydrase | JN244441 | *Homo sapiens* | 10^-42^ | 8 |
| transferrin | JN244447-48 | *Saccoglossus kowalevskii* | 10^-33^ | 5 |
| adenosylmethionine synthetase | JN244451 | *Nematostella vectensis* | 10^-164^ | 3 |
| oligosaccharyltransferase | JN244450 | *Nematostella vectensis* | 0 | 2 |
| inorganic pyrophosphatase | JN244445 | *Nematostella vectensis* | 10^-114^ | 2 |
| arsenic methyltransferase | JN244439 | *Danio rerio* | 10^-82^ | 2 |
| 1,4-α-glucan branching enzyme | JN244461 | *Nematostella vectensis* | 0 | 1 |
| fructose 1,6 bisphosphate aldolase | JN244456 | *Nematostella vectensis* | 10^-146^ | 1 |
| hydantoinase/oxoprolinase | JN244458 | *Branchiostoma floridae* | 10^-127^ | 1 |
| S-adenosylmethionine-dependent methyltransferase | JN244440 | *Nematostella vectensis* | 10^-119^ | 1 |
| phosphaditylserine decarboxylase proenzyme | JN244454 | *Nematostella vectensis* | 10^-110^ | 1 |
| arylsulfatase | JN244457 | *Salpingoeca* sp. | 10^-87^ | 1 |
| abhydrolase domain-containing protein | JN244455 | *Nematostella vectensis* | 10^-77^ | 1 |
| dTDP-D-glucose 4,6-dehydratase | JN244460 | *Nematostella vectensis* | 10^-73^ | 1 |
| 6-phosphofructokinase | JN244463 | *Callithrix jacchus* | 10^-71^ | 1 |
| ferritin | JN244446 | *Nematostella vectensis* | 10^-70^ | 1 |
| long chain fatty acid CoA ligase | JN244464 | *Ornithorhynchus anatinus* | 10^-68^ | 1 |
| cytochrome b5 reductase | JN244443 | *Nematostella vectensis* | 10^-66^ | 1 |
| 4-amino-4-deoxy-L-arabinose transferase | JN244465 | *Nematostella vectensis* | 10^-45^ | 1 |
| alanine dehydrogenase | JN244459 | *Nematostella vectensis* | 10^-34^ | 1 |
| N-acetyltransferase 8 | JN244452 | *Nematostella vectensis* | 10^-32^ | 1 |
| cytochrome c oxidase subunit VI | JN244444 | *Trichoplax adhaerens* | 10^-24^ | 1 |
| transketolase-like protein | JN244462 | *Harpegnathos saltator* | 10^-20^ | 1 |
| 3-ketoacyl-(acyl-carrier-protein) reductase | JN244453 | *Nematostella vectensis* | 10^-11^ | 1 |
| EDRK rich factor | JN244449 | *Ciona intestinalis* | 10^-7^ | 1 |

| ***S. hystrix* gene NCBI accession Top tBLASTx hit (species) e value clones (#)** | | | | |
| --- | --- | --- | --- | --- |
| **MITOCHONDRIAL** | | | | |
| tRNA-methionine and 28s | JN244466 | *Seriatopora hystrix* | 10^-166^ | 35 |
| NADH dehydrogenase and ATP synthase | JN244467 | *Seriatopora hystrix* | 10^-172^ | 4 |
| mitochondrial solute carrier | JN244472 | *Nematostella vectensis* | 10^-124^ | 4 |
| mitochondrial solute carrier 25 | JN244475 | *Nematostella vectensis* | 10^-145^ | 1 |
| ganglioside-induced differentiation-associated protein 2 | JN244473 | *Nematostella vectensis* | 10^-82^ | 1 |
| mitochondrial ATP synthase | JN244474 | *Nematostella vectensis* | 10^-67^ | 1 |
| mitochondrial ribosomal subunit S27 | JN244470 | *Nematostella vectensis* | 10^-23^ | 1 |
| **PROTEIN HOMEOSTASIS** | | | | |
| WD40 | JN244499 | *Nematostella vectensis* | 10^-56^ | 7 |
| cathepsin peptidase | JN244485 | *Trichoplax adhaerens* | 10^-110^ | 2 |
| ring finger protein | JN244487 | *Nematostella vectensis* | 10^-71^ | 2 |
| MPN-RPN7 proteasome protein | JN244486 | *Nematostella vectensis* | 10^-10^ | 2 |
| dynein intermediate chain 1 | JN244491 | *Nematostella vectensis* | 0 | 1 |
| alanine-glyoxylate aminotransferase | JN244494 | *Nematostella vectensis* | 10^-138^ | 1 |
| palmitoyl protein thioesterase | JN244496 | *Nematostella vectensis* | 10^-128^ | 1 |
| proteasome subunit β type-4 | JN244489 | *Nematostella vectensis* | 10^-99^ | 1 |
| proteasome subunit α type-2 | JN244492 | *Nematostella vectensis* | 10^-88^ | 1 |
| ATP-dependent metalloprotease | JN244490 | *Nematostella vectensis* | 10^-76^ | 1 |
| proteasome subunit β type-2 | JN244497 | *Nematostella vectensis* | 10^-70^ | 1 |
| ubiquitin-conjugating enzyme | JN244498 | *Strongylocentrotus purpuratus* | 10^-44^ | 1 |
| interferon gamma-inducible protein 30 | JN244495 | *Nematostella vectensis* | 10^-37^ | 1 |
| SEC11-like | JN244493 | *Saccoglossus kowalevskii* | 10^-7^ | 1 |
| **RECEPTORS/MEMBRANE PROTEINS** | | | | |
| guanine nucleotide binding protein | JN244505 | *Nematostella vectensis* | 10^-161^ | 2 |
| somatin-like protein 2 | JN244507 | *Trichoplax adhaerens* | 10^-114^ | 1 |
| BTB/POZ domain-containing protein | JN244511 | *Nematostella vectensis* | 10^-88^ | 1 |
| integrin β2 | JN244510 | *Acropora millepora* | 10^-78^ | 1 |
| receptor expression-enhancing protein 5 | JN244508 | *Nematostella vectensis* | 10^-72^ | 1 |
| Rho GDP-dissociation inhibitor | JN244506 | *Saccoglossus kowalevskii* | 10^-46^ | 1 |
| G-protein coupled receptor | JN244503-04 | *Saccoglossus kowalevskii* | 10^-26^ | 1 |
| cubilin precursor | JN244501 | *Mus musculus* | 10^-23^ | 1 |
| mannose receptor | JN244509 | *Branchiostoma floridae* | 10^-15^ | 1 |
| **SIGNAL TRANSDUCTION** | | | | |
| TNF receptor-associated factor 3 | JN244552 | *Nematostella vectensis* | 10^-36^ | 3 |
| arginine kinase | JN244540 | *Nematostella vectensis* | 10^-159^ | 2 |
| phosphatase 1 | JN244539 | *Nematostella vectensis* | 10^-50^ | 2 |
| adenosine kinase | JN244545 | *Nematostella vectensis* | 10^-148^ | 1 |
| tyrosine-protein phosphatase | JN244546 | *Nematostella vectensis* | 10^-157^ | 1 |
| cAMP-dependent protein kinase | JN244543 | *Nematostella vectensis* | 10^-124^ | 1 |
| cyclin-dependent kinase 7 | JN244544 | *Nematostella vectensis* | 10^-97^ | 1 |
| protein kinase C inhibitor protein 1 | JN244549 | *Ovis aries* | 10^-79^ | 1 |
| serine/threonine-protein kinase | JN244542 | *Nematostella vectensis* | 10^-65^ | 1 |
| suppressor of cytokine signaling | JN244548 | *Nematostella vectensis* | 10^-36^ | 1 |
| protein tyrosine phosphatase | JN244541 | *Nematostella vectensis* | 10^-14^ | 1 |
| TNF-receptor associated factor 4 | JN244547 | *Trichoplax adhaerens* | 10^-14^ | 1 |
| ***S. hystrix* gene NCBI accession Top tBLASTx hit (species) e value clones (#)** | | | | |
| **STRESS** | | | | |
| ***heat shock protein/cognate 70* (*hsp/c)*** | HM147130 | *Seriatopora hystrix* | 0 | 1 |
| allograft inflammatory factor | JN244550 | uncultured cnidarian | 10^-57^ | 1 |
| universal stress protein | JN244554 | *Nematostella vectensis* | 10^-32^ | 1 |
| DnaJ homolog | JN244555 | *Hydra magnipapillata* | 10^-12^ | 1 |
| **STRUCTURAL** | | | | |
| beta gamma crystallin | JN244558 | *Montipora capitata* | 10^-50^ | 26 |
| collagen | JN244560 | *Nematostella vectensis* | 10^-64^ | 2 |
| collagen α1, type II | JN244561 | *Nematostella vectensis* | 10^-48^ | 2 |
| collagen, α1, type II | JN244559 | *Nematostella vectensis* | 10^-22^ | 2 |
| fibronectin type 3 w/ ankyrin repeat domain | JN244566 | *Nematostella vectensis* | 10^-97^ | 1 |
| collagen, α2, type I | JN244563 | *Nematostella vectensis* | 10^-59^ | 1 |
| ankyrin-1 | JN244556 | *Nomascus leukogenys* | 10^-38^ | 1 |
| collagen, α1, type III | JN244564 | *Mus musculus* | 10^-32^ | 1 |
| collagen, α2, type XI | JN244562 | *Hydra magnipapillata* | 10^-17^ | 1 |
| ankyrin | JN244557 | *Strongylocentrotus purpuratus* | 10^-9^ | 1 |
| **TRAFFICKING** | | | | |
| rab24-like | JN244569 | *Nematostella vectensis* | 10^-13^ | 4 |
| intraflagellar transport protein | JN244568 | *Nematostella vectensis* | 0 | 2 |
| vacuolar sorting-associated protein 26 | JN244579 | *Branchiostoma floridae* | 10^-116^ | 2 |
| myoferlin | JN244567 | *Nematostella vectensis* | 10^-167^ | 1 |
| importin subunit α7 | JN244573 | *Branchiostoma floridae* | 10^-139^ | 1 |
| archain 1 | JN244574 | *Nematostella vectensis* | 10^-115^ | 1 |
| rab7 | JN244570 | *Nematostella vectensis* | 10^-107^ | 1 |
| translocon-associated protein gamma | JN244576 | *Nematostella vectensis* | 10^-74^ | 1 |
| RAS oncogene family-like 4 | JN244571 | *Nematostella vectensis* | 10^-70^ | 1 |
| clathrin light chain A | JN244572 | *Nematostella vectensis* | 10^-67^ | 1 |
| synaptotagmin-like protein 2 | JN244575 | *Nematostella vectensis* | 10^-65^ | 1 |
| vesicle-trafficking protein sec22a | JN244577 | *Nematostella vectensis* | 10^-32^ | 1 |
| coatomer subunit gamma-2 | JN244578 | *Nematostella vectensis* | 10^-14^ | 1 |
| **TRANSCRIPTION** | | | | |
| ribonucleotide reductase | JN244583 | *Urechis caupo* | 10^-152^ | 2 |
| pre-mRNA-splicing regulator WTAP | JN244592 | *Nematostella vectensis* | 10^-73^ | 2 |
| Taf3 protein | JN244581 | *Nematostella vectensis* | 10^-50^ | 2 |
| dicer 1 | JN244590 | *Nematostella vectensis* | 10^-68^ | 1 |
| DREV methyltransferase | JN244582 | *Strongylocentrotus purpuratus* | 10^-54^ | 1 |
| reverse transcriptase | JN244587 | *Nematostella vectensis* | 10^-50^ | 1 |
| BR140-related | JN244580 | *Saccoglossus kowalevskii* | 10^-43^ | 1 |
| transcription elongation factor B | JN244584 | *Nematostella vectensis* | 10^-41^ | 1 |
| U6 snRNA-associated Sm-like protein LSm7 | JN244586 | *Nematostella vectensis* | 10^-40^ | 1 |
| splicing factor U2AF 65 kDa subunit | JN244588 | *Nematostella vectensis* | 10^-34^ | 1 |
| histone deacetylase | JN244585 | *Nematostella vectensis* | 10^-18^ | 1 |
| ATP-dependent RNA helicase Ddx1 | JN244589 | *Camponotus floridanus* | 10^-7^ | 1 |

| ***S. hystrix* gene NCBI accession Top tBLASTx hit (species) e value clones (#)** | | | | | |
| --- | --- | --- | --- | --- | --- |
| **TRANSCRIPTION FACTOR** | | | | | |
| CCAAT/enhancer binding protein (I) | | JN244594 | *Nematostella vectensis* | 10^-18^ | 52 |
| bZIP transcription factor 1 | | JN244596 | *Nematostella vectensis* | 10^-27^ | 2 |
| CCAAT/enhancer binding protein (II) | | JN244595 | *Nematostella vectensis* | 10^-15^ | 2 |
| jun-like transcription factor | | JN244597 | *Daphnia pulex* | 10^-9^ | 2 |
| TGF- β type I receptor | | JN244598 | *Ornithorhynchus anatinus* | 10^-70^ | 1 |
| bromo-tif1-like | | JN244604 | *Nematostella vectensis* | 10^-57^ | 1 |
| transcription factor Elk-like | | JN244601 | *Nematostella vectensis* | 10^-33^ | 1 |
| transcription factor HES-4-A | | JN244605 | *Nematostella vectensis* | 10^-27^ | 1 |
| transcription factor AP-1 | | JN244603 | *Nematostella vectensis* | 10^-25^ | 1 |
| cAMP-responsive element-binding protein-like 2 | | JN244599 | *Nematostella vectensis* | 10^-22^ | 1 |
| ETS transcription factor | | JN244600 | *Nematostella vectensis* | 10^-18^ | 1 |
| I-kappa-B | | JN244602 | *Nematostella vectensis* | 10^-67^ | 1 |
| **TRANSLATION** | | | | | |
| elongation factor 1α | JN244606 | | *Pocillopora damicornis* | 0 | 4 |
| glutamyl-polyl tRNA synthetase | JN244611 | | *Canis familiaris* | 10^-169^ | 1 |
| polyadenylate-binding protein 1 | JN244610 | | *Camponotus floridanus* | 10^-144^ | 1 |
| translation initiation factor 2 | JN244607 | | *Nematostella vectensis* | 10^-127^ | 1 |
| methionyl aminopeptidase 2 | JN244609 | | *Nematostella vectensis* | 10^-118^ | 1 |
| RNA binding motif protein 34 | JN244608 | | *Nematostella vectensis* | 10^-36^ | 1 |
| **TRANSPORT** | | | | | |
| voltage-dependent calcium channel | JN244618 | | *Nematostella vectensis* | 10^-132^ | 1 |
| solute carrier family 44, member 2 | JN244616 | | *Nematostella vectensis* | 10^-73^ | 1 |
| cation efflux protein (I)* | HM191477 | | *Nematostella vectensis* | 10^-68^ | 1 |
| cation efflux protein (II) | JN244612 | | *Nematostella vectensis* | 10^-46^ | 1 |
| solute carrier family 17, member 9-like | JN244615 | | *Nematostella vectensis* | 10^-46^ | 1 |
| organic anion transporter polypeptide | JN244614 | | *Nematostella vectensis* | 10^-20^ | 1 |
| transient receptor potential cation channel (I)* | HM147132 | | *Danio rerio* | 10^-18^ | 1 |
| calcium-activated potassium channel | JN244619 | | *Nematostella vectensis* | 10^-13^ | 1 |
| transient receptor potential cation channel (II) | JN244613 | | *Nematostella vectensis* | 10^-6^ | 1 |
