# Supplementary material for: Assessing the Impacts of Experimentally Elevated Temperature on the Biological Composition and Molecular Chaperone Gene Expression of a Reef Coral
Source: PLoS One. 2011 Oct 27;6(10):e26529. doi: 10.1371/journal.pone.0026529 (PMC3203140; doi:10.1371/journal.pone.0026529)
Supplement: Table S2 — Real-time quantitative PCR primers designed for use with SYBR® green I chemistry with whole holobiont total RNA extracts from Seriatopora hystrix . Assays in which it was necessary to dilute the cDNA 10-fold in order to achieve optimal results are denoted by “a.” For all assays, each cycle included a 95°C hold for 15 s, followed by a 60 s incubation at the respective annealing temperature. (DOCX) [file pone.0026529.s002.docx]

| Gene name  (fragment length) | Compartment | Forward primer (‘5-3’) | Reverse primer (‘5-3’) | [primer] (nM) | Annealing temp. (°C) | Cycle (#) |
| --- | --- | --- | --- | --- | --- | --- |
|  |  |  |  |  |  |  |
| ascorbate peroxidase (107 bp) | *Symbiodinium* | GCCAAGTTCAAGGAGCATGTA | AGCTGACCACATCCCAACT | 150 | 61 | 40 |
|  |  |  |  |  |  |  |
| nitrate transporter  (97 bp) | *Symbiodinium* | CCACCCATTTCAGGACCTAT | CCAGGGACCTAGCAAACAA | 200 | 62 | 40 |
|  |  |  |  |  |  |  |
| photosystem I, subunit III  (136 bp) | *Symbiodinium* | GTGGAGTTGACATTGACTTGGA | TGCTGCTTGGTGGTCTTGTA | 500 | 59 | 35 |
|  |  |  |  |  |  |  |
| ezrin  (162 bp)^a^ | *S. hystrix* | CAGCGGAAAATGCTTCAGC | TTCATCAACCCTCTGCTTAGTG | 400 | 59 | 35 |
|  |  |  |  |  |  |  |
| phospholipase α2 (174 bp) | *S. hystrix* | GCAAACGGACGGAATCAAAGA | GGCATCTTCAAACACGGAGA | 100 | 60 | 40 |
|  |  |  |  |  |  |  |
| transient receptor cation channel  (114 bp) | *S. hystrix* | TGGTCGGTTTGGCTGTTG | TCCATTGTGGGAGAGATTCTTC | 250 | 60 | 31 |
|  |  |  |  |  |  |  |
| organic anion transporter  (130 bp) | *S. hystrix* | ACGGTTGGCTGTTTGACACT | AACACATGGCAAGTTCCGG | 200 | 60 | 35 |

**Table S2**. Real-time quantitative PCR primers designed for use with SYBR® green I chemistry with whole holobiont total RNA extracts from *Seriatopora hystrix*. Assays in which it was necessary to dilute the cDNA 10-fold in order to achieve optimal results are denoted by “^a^.” For all assays, each cycle included a 95°C hold for 15 s, followed by a 60 s incubation at the respective annealing temperature.
